# Biosurfactants Produced by Marine Microorganisms with Therapeutic Applications

**DOI:** 10.3390/md14020038

**Published:** 2016-02-18

**Authors:** Eduardo J. Gudiña, José A. Teixeira, Lígia R. Rodrigues

**Affiliations:** CEB—Centre of Biological Engineering, University of Minho, 4710-057 Braga, Portugal; jateixeira@deb.uminho.pt (J.A.T.); lrmr@deb.uminho.pt (L.R.R.)

**Keywords:** biosurfactant, antimicrobial activity, anti-adhesive activity, anti-biofilm activity, anti-cancer activity, metagenomics

## Abstract

Marine microorganisms possess unique metabolic and physiological features and are an important source of new biomolecules, such as biosurfactants. Some of these surface-active compounds synthesized by marine microorganisms exhibit antimicrobial, anti-adhesive and anti-biofilm activity against a broad spectrum of human pathogens (including multi-drug resistant pathogens), and could be used instead of existing drugs to treat infections caused by them. In other cases, these biosurfactants show anti-cancer activity, which could be envisaged as an alternative to conventional therapies. However, marine biosurfactants have not been widely explored, mainly due to the difficulties associated with the isolation and growth of their producing microorganisms. Culture-independent techniques (metagenomics) constitute a promising approach to study the genetic resources of otherwise inaccessible marine microorganisms without the requirement of culturing them, and can contribute to the discovery of novel biosurfactants with significant biological activities. This paper reviews the most relevant biosurfactants produced by marine microorganisms with potential therapeutic applications and discusses future perspectives and opportunities to discover novel molecules from marine environments.

## 1. Introduction

A huge and extensive source of natural compounds can be retrieved from the marine environment [1,2]. Marine microorganisms exhibit unique metabolic and physiological capabilities conferring them the ability to survive in extreme conditions and consequently produce novel metabolites that cannot be found elsewhere [3,4]. Hence, the marine environment holds a great promise towards the discovery of novel bioactive and relevant compounds including antibiotics, enzymes, vitamins, drugs and biosurfactants, among others [1,5,6].

Biosurfactants have attracted much attention in recent years; they are surface-active compounds synthesized by microorganisms that exhibit diverse chemical structures, including glycolipids, lipopeptides, polysaccharide-protein complexes, phospholipids, fatty acids and neutral lipids [4,5,7]. Due to their amphipathic nature, biosurfactants display a variety of surface activities, which allows their application in several fields related with emulsification, foaming, detergency, wetting, dispersion and solubilisation of hydrophobic compounds [8,9]. Many biosurfactants have been reported to possess a similar or better performance when compared with synthetic surfactants, which in addition to their lower toxicity, higher biodegradability and effectiveness at extreme temperatures, salinities and pH values, make them a green alternative to their chemical counterparts in different applications, including agriculture, food, cosmetics or petroleum industries, as well as in bioremediation [8,9,10,11,12]. Furthermore, several biosurfactants exhibit antibacterial, antifungal, antiviral or anti-tumour activities, making them potential alternatives to conventional therapeutic agents in many biomedical applications [4,7,8,9,13].

Biosurfactant-producing microorganisms are ubiquitous, inhabiting both water (sea, fresh water and ground water) and land (soil, sediment and sludge), as well as environments characterized by extreme conditions of pH, temperature or salinity (e.g., hyper saline sites and oil reservoirs) [4,14,15,16,17]. Due to their unique environmental conditions, the marine environments are a good source for the isolation of new biosurfactant-producing microorganisms. A considerable number of marine microorganisms able to produce biosurfactants with different structures have been reported, as discussed below. However, it has to be taken into account that the great majority of the marine microbial diversity remains unexplored, mainly due to the difficulty of growing marine microorganisms under laboratory conditions [6,18,19,20].

This paper reviews the most relevant biosurfactants produced by marine microorganisms with potential therapeutic applications, and discusses future perspectives and opportunities to discover novel biosurfactants from marine environments.

## 2. Biosurfactants from Marine Microorganisms: New Weapons to Fight Human Pathogens

Most human bacterial infections can be successfully treated using current antibiotic therapies. However, in recent years, a significant increase in the emergence of pathogenic microorganisms resistant to the available antimicrobials has been observed, including multi-drug resistant (MDR) pathogens, which has been associated with the misuse or abuse of antibiotics. As a result, persistent and difficult to treat infectious diseases appeared, which constitutes a serious public health problem [21]. Furthermore, in the last decades, the discovery of new antimicrobials has declined considerably (only two new classes of antibiotics have been commercialized since 1962), due to the difficulties in identifying novel and effective compounds and the subsequent high economic investments required for their development [21]. Therefore, there is an urgent demand for novel antimicrobial drugs.

Several biosurfactants have been reported to exhibit antimicrobial activity against different human pathogens; furthermore, these compounds usually display anti-adhesive and anti-biofilm activities, making them useful to reduce the adhesion and colonization by pathogenic microorganisms, as well as to remove pre-formed biofilms [22,23,24,25]. Although biosurfactants have been extensively studied, most of them were obtained from microorganisms isolated from terrestrial samples or hydrocarbon-polluted areas, whereas biosurfactants produced by marine microorganisms have been less explored [26]. The most relevant biosurfactants produced by marine microorganisms exhibiting antimicrobial, anti-adhesive or anti-biofilm activities against different pathogenic and opportunistic microorganisms are shown in Table 1. Several of these biosurfactants are effective against a broad spectrum of human pathogens, including Gram-positive and Gram-negative bacteria, as well as the yeast *Candida albicans*. Furthermore, in some cases they are also effective against MDR clinical isolates. Therefore, they can be an alternative to the existing drugs to treat infections caused by those pathogens.

It should be noted that, with the exception of the isolate *Serratia marcescens* described by Dusane and co-workers [28], all the other microorganisms reported in Table 1 were isolated from marine samples collected in the coastal waters of India, which suggests a higher investment of this country in the exploration of the marine resources. Regarding the phylogeny of the different isolates, more than half of them are actinomycetes. Furthermore, most of those microorganisms were isolated from marine macro-organisms. Marine sponges and other marine invertebrates are important sources of novel bioactive compounds, including antimicrobial, anti-adhesive and anti-biofilm agents. These compounds play a critical role in their defence against predators, infectious agents and biofilm-forming microorganisms, and most of them are synthesized by symbiotic microorganisms and not by the host macro-organism [28,31,37,38].

In most of the above referred studies, the biosurfactants were only partially characterized and their activities were studied using the crude extracts rather than purified molecules. However, in other cases a more complete chemical characterization was performed. Regarding the lipopeptide biosurfactant produced by the *Bacillus circulans* strain reported by Das *et al.* [32], six different fractions were obtained from the crude extract using reverse phase HPLC, and only one of them was responsible for the antimicrobial activity exhibited by the crude biosurfactant. It should be pointed out that, contrary to other lipopeptide biosurfactants such as surfactin, this biosurfactant did not show haemolytic activity, which could facilitate its use as a therapeutic agent [32]. Similarly, the lipopeptide biosurfactant produced by *B. circulans* DMS-2 was identified as a mixture of different fengycin isoforms (including C_15_-, C_16_- and C_17_-fengycin). Four different surface-active fractions were resolved and purified through HPLC, and only one of them (containing C_16_- and C_17_-fengycin) was responsible for the antimicrobial activity observed in the crude biosurfactant [34].

Additionally, several biosurfactants reported in Table 1 displayed a similar or better performance when compared with conventional antibiotics. In order to compare the antimicrobial activity exhibited by different compounds, the minimum inhibitory concentration (MIC) and minimum bactericidal concentration (MBC) are commonly used parameters. The MIC for an antimicrobial compound against a specific microorganism is the minimum concentration of that compound that completely inhibits the growth of that microorganism. The MBC is the minimum concentration of that compound that is lethal for that microorganism. One of the HPLC-purified fractions of the lipopeptide biosurfactant produced by *B. circulans* showed lower MICs and MBCs against *S. marcescens*, *Proteus vulgaris* and *Enterobacter cloacae* (between 10 and 60 µg·mL^−1^) when compared with the conventional antibiotics penicillin and streptomycin (between 40 and 900 µg·mL^−1^). Regarding the MDR *Escherichia coli*, *Klebsiella pneumoniae* and *Staphylococcus aureus* (which were resistant to penicillin and streptomycin at concentrations up to 1000 µg·mL^−1^), MICs and MBCs between 60 and 800 µg·mL^−1^ and 200–1000 µg·mL^−1^, respectively, were obtained for this biosurfactant [32]. The glycolipid biosurfactant produced by *Streptomyces* sp. MAB36 possessed a similar inhibitory activity against *Aspergillus niger* and *C. albicans* to the conventional antifungal nystatin [30]. The biosurfactant produced by *Nocardiopsis dassonvillei* MAD08 was more effective against *E. coli* and *Staphylococcus epidermidis* than chloramphenicol [38]. Finally, the biosurfactant produced by *S. marcescens* exhibited a higher inhibitory effect against *C. albicans* and *Pseudomonas aeruginosa* as compared to the conventional antimicrobials fluconazole and streptomycin, respectively [28].

In addition, some of these biosurfactants displayed a considerable anti-adhesive and anti-biofilm activity. The biosurfactant produced by *B. circulans* (partially purified through gel filtration chromatography) exhibited anti-adhesive activity at concentrations between 0.1 and 10 mg·mL^−1^. At the highest concentration tested (10 mg·mL^−1^), microbial adhesion was inhibited between 84% and 89%, and pre-formed biofilms were removed (with efficiencies between 59% and 94%) for all the pathogenic microorganisms tested [33]. The glycolipid biosurfactant produced by *Brevibacterium casei* MSA19 (partially purified through thin layer chromatography) removed pre-formed biofilms of all the pathogenic microorganisms tested at 30 µg·mL^−1^ [27].

Despite their potential applications, the widespread use of biosurfactants is still limited by their low productivities. The optimization of the culture medium and cultivation conditions can greatly contribute to increase their production yields [31,37]. Furthermore, the composition of the culture medium can alter the structure and activity of the biosurfactant. In the case of the isolate *B. circulans*, the antimicrobial activity of the biosurfactant was dependent on the carbon source used, due to the production of different isoforms in the different media; the biosurfactant produced using culture media containing glycerol, starch or sucrose exhibited a higher antimicrobial activity when compared with the one produced in a medium containing glucose [39]. However, in the case of marine microorganisms, which are adapted to the marine environment conditions, their cultivation in the laboratory or in industrial fermenters can be difficult. One alternative is to produce those biosurfactants in heterologous hosts. In the case of the lipopeptide biosurfactant produced by *Bacillus licheniformis* NIOT-AMKV06, three genes involved in its biosynthesis (*sfp*, *sfpO*, and *srfA*) were cloned and expressed in *E. coli*. As a result, biosurfactant production was increased from 3 g·L^−1^ up to 11.7 g·L^−1^ [35].

Although not included in Table 1, the strain *Streptomyces* sp. ISP2-49E, isolated from marine sediment samples obtained from Galveston Bay (Texas) must be mentioned. This isolate produced the rhamnolipid biosurfactant l-rhamnosyl-l-rhamnosyl-β-hydroxydecanoyl-β-hydroxydecanoate (Rha-Rha-C_10_-C_10_), being the first report on a rhamnolipid-producing *Streptomyces* strain [15]. Although the properties of the biosurfactant synthesized by this isolate were not studied in detail, rhamnolipids have been reported to possess a broad spectrum of antimicrobial and anti-adhesive activities [22,23]. However, the main rhamnolipid producers are *P. aeruginosa* strains, an opportunistic human pathogen. Therefore, the use of alternative non-pathogenic rhamnolipid producers can contribute to the safe use of rhamnolipids as therapeutic agents. That can be achieved using either non-pathogenic natural rhamnolipid-producing strains [15,40,41,42], or engineered non-pathogenic hosts expressing the genes required for the synthesis of rhamnolipids [43,44].

The antagonistic activities exhibited by these biosurfactants against human pathogens (including MDR pathogens) make them candidates to be used as an alternative to traditional antibiotics. However, despite their great potential, none of these compounds is yet being used for the treatment of human infections.

## 3. Biosurfactants from Marine Microorganisms: Alternative Anti-Cancer Agents

Cancer represents an extremely important health risk affecting millions of people worldwide [45]; hence, any progress leading to enhanced survival is a global priority. Given its unpredictable nature, cancer is a major concern for human health. Several strategies have been pursued over the years, whether searching for new biomarkers, treatments or drugs. However, despite these efforts, a successful targeted selective and non-toxic therapy is still to be developed. Traditional cancer chemotherapy has mainly been based on using highly cytotoxic drugs that non-specifically target any dividing cells; this may result in a modest improvement in patient survival, thus overall prognosis of most patients remains dismal and treatment is non-specific, non-selective and toxic. In this sense, the search and development of new anti-cancer drugs that can overcome the multi-drug resistance of cancer cells remains a great challenge. Currently, many anti-cancer drugs used in clinical practice are natural products or derivatives thereof [46,47,48,49]. For that reason, it is likely that the continued and systematic exploration of natural sources, such as the marine microbiota, will lead to different and unforeseen compounds with interesting biological activities, including anti-cancer activity [50,51]. The microbial production of anti-cancer drugs is advantageous compared to their extraction from natural sources such as plants, namely the possibility of genetically engineering microbes for a given purpose, as well as their higher production rates [52]. As previously mentioned, biosurfactants are among those microbial compounds exhibiting promising biological activities [53].

**Figure 1:**
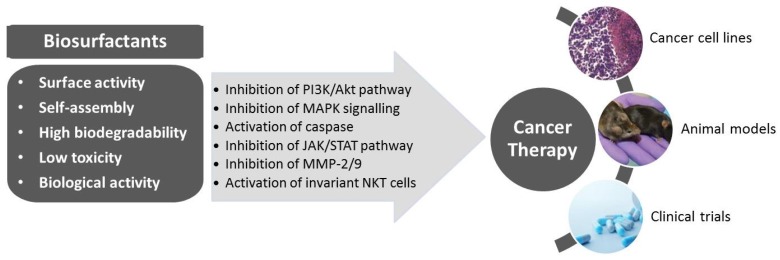
Properties and anti-cancer effects of biosurfactants towards the design of novel cancer therapies.

Biosurfactants, in particular lipopeptides and glycolipids, have been highlighted for their potential to be used as anti-cancer agents interfering with cancer progression processes (Figure 1) [7]. These compounds have been implicated in several intercellular molecular recognition steps comprising signal transduction, cell differentiation and cell immune response, among others [24]. In addition, they exhibit low toxicity, high efficacy and easy biodegradability, which are relevant features in any anti-cancer agent. Different mechanisms underlying the anti-cancer activity of biosurfactants have been proposed including the delay of cell cycle progression; inhibition of crucial signalling pathways such as Akt, extracellular signal-regulated kinase/c-Jun N-terminal kinase (ERK/JNK) and Janus kinase/signal transducer and activator of transcription (JAK/STAT); reduction of angiogenesis; activation of natural killer T (NKT) cells; and induction of apoptosis through death receptors in cancer cells. In addition, the ability of biosurfactants to disrupt cell membranes, leading to a sequence of events that include lysis, increased membrane permeability and metabolite leakage, has also been pointed as a probable mechanism of anti-cancer activity [54].

Despite this exciting potential and the great diversity of chemical structures that can be found among biosurfactants, the majority of the studies on their anti-cancer activity have been conducted with few well-known molecules produced by microbes mainly isolated from terrestrial sources. Therefore, other natural environments, such as the marine microbiota, open up a great opportunity to discover new biosurfactants exhibiting distinct chemical structures and powerful anti-cancer activities provided by different mechanisms of action and/or different targets.

Lipopeptides, particularly surfactin, have been widely studied for their potential anti-cancer activity against a number of cancer cell lines [7,53,55]. The anti-cancer activity of surfactin has been related with the hydrophobic nature of the fatty acid moiety that interacts with the acyl chain of membrane-bound phospholipids [56]. Simultaneously, its peptide moiety strongly interacts with the polar heads of the membrane lipids in cancer cells. Surfactin, holding a longer fatty acid chain, penetrates more efficiently into the cancer cell membrane [56]. Different mechanisms have been suggested for its anti-cancer activity depending on the cancer models evaluated (breast, colon, leukaemia, hepatic, melanoma) [7] including the inhibition of matrix metalloproteinases (protease enzymes involved in invasion and metastasis processes) [57]; PI3/Akt and MAPK signalling pathways [56]; cell cycle arrest at G2/M [58]; and induction of apoptosis via ROS/JNK-mediated mitochondrial/caspase pathway [59]. This cyclic lipopeptide comprised by seven amino acids and a lipid moiety (containing 13 to 15 carbons) is produced by several strains of bacilli retrieved from different sources, including the marine environment [7,53,60]. The marine bacterium *B. circulans* DMS-2 was found to produce lipopeptides, namely surfactin and fengycin isoforms, displaying a significant and selective anti-proliferative activity against the human colon cancer cell lines HCT-15 (IC_50_ 80 µg·mL^−1^) and HT-29 (IC_50_ 120 µg·mL^−1^) [55].

In addition, different bacilli strains produce iturins (bacillomycins, iturin A/C and mycosubtilins). These lipopeptides are amphiphilic molecules containing a cyclic peptide chain conjugated with a β-amino fatty acid (containing 13 to 17 carbons). Iturin A, produced by a marine *Bacillus megaterium* strain, was found to significantly impair proliferation and inhibit the Akt signalling network leading to apoptosis induction in breast cancer cells (MDA-MB-231 and MCF-7). It is important to note that treatments that can inhibit breast cancer types exhibiting aberrant Akt activity are of utmost importance. Additionally, this biosurfactant inhibited EGF induced Akt phosphorylation and its downstream targets GSK3β and FoxO3α. Iturin A was also found to inhibit tumour growth in a breast cancer xenograft model [61]. Other biosurfactants produced by marine microorganisms have been reported among the iturin class, such as hallobacillin [62] and mixirins [63]. Hallobacillin, produced by a *Bacillus* sp. isolated from marine sediments near the Guaymas Basin (Mexico), is cytotoxic against the human colon cancer cell line HCT-116 (IC_50_ 0.98 µg·mL^−1^) [63]. Mixirins (A, B and C), also isolated from a marine *Bacillus* sp. strain, are cyclic octapeptides comprised by a mixture of l- and d-amino acid with an unusual β-amino alkanoic acid. Likewise, these lipopeptides were cytotoxic against colon cancer cells, being the variant A the most potent [63].

It is clear that the marine environment represents a promising source of novel added value compounds. Among these compounds, some new biosurfactant structures have been reported, namely somocystinamide A [64], fellutamides [65,66,67], rakicidin [68,69,70,71,72] and apratoxin [73,74,75,76,77].

Wrasidlo and collaborators [78] obtained the lipopeptide somocystinamide A from the cyanobacteria *Lyngbya majuscula*. This biosurfactant exhibited significant cytotoxicity against leukaemia, lung, breast and prostate cancer cells with IC_50_ values ranging from 1.3 µM to 970 nM depending on the cancer model. It is considered a pluripotent inhibitor of angiogenesis and tumour cell proliferation. Moreover, it induced apoptosis in Jurkat and leukaemia cells through caspase-8 activation and PARP cleavage. Somocystinamide A was also found to effectively block endothelial cell tube formation *in vitro* and blood vessel growth in a zebrafish model, thus suggesting its anti-angiogenic character.

Fellutamides A and B are linear lipopeptides isolated from the fish-derived fungus *Penicillium fellutanum*. These biosurfactants were found to be cytotoxic against P388, L1210 murine leukaemia cells and KB human epidermoid carcinoma cells [67]. Fellutamides C and F, isolated from the sponge-derived fungus *Aspergillus versicolor*, displayed cytotoxic effects against SK-MEL-2 skin cancer, XF498 CNC cancer, HCT-15 colon cancer, A549 lung cancer and SK-OV-3 ovarian cancer cell lines, with IC_50_ values ranging from 3.1 to 33.1 µM for fellutamide C, and between 0.2 and 3.1 µM for fellutamide F [65,66].

Rakicidins are anti-cancer lipopeptides produced by the marine bacterium *Micromonospora* [69,70]. Among these lipopeptides, rakicidin A exhibits a unique hypoxia-selective cytotoxicity against several cancer cell lines, such as HCT-8 and PANC-1 [72]. Hypoxia is present mainly in solid tumours and is associated with a poor prognosis and clinical outcome since it triggers invasiveness, angiogenesis, metastasis and apoptosis evasion [72]. Rakicidin B has also been reported to be active against oesophageal squamous carcinoma cells (EC109), lung cancer cells (A549 and 95D), gastric cancer cells (SGC7901), uterine cervix cancer cells (HeLa) and hepatocellular carcinoma cells (HepG2) [70]. This rakicidin derivative induced apoptosis through the activation of caspase-3, -7 and -9, and blocked MAPK and JNK/p38 signalling pathways. Rakicidin derivatives C and D containing short lipid chains were non cytotoxic [68], although the derivative D was found to interfere with the invasiveness of aggressive breast cancer cells [68].

Apratoxins (derivatives A to G) are a new group of cyclic lipopeptides isolated from marine cyanobacteria [74] that exhibit a significant cytotoxicity against a number of cancer cells. Apratoxin A induced apoptosis through caspases activation and inhibited the IL-6 signalling pathway in human bone osteosarcoma U2OS cells. Two apratoxin analogues (apratoxin A sulfoxide and apratoxin H) isolated from the cyanobacterium *Moorea producens* showed a great cytotoxicity on human NCI-H460 lung cancer cells [76]. Other research groups also reported strong cytotoxicity against this cell line using other apratoxin derivatives (D, F and G) [75]. Apratoxin E, F and G were also active against HCT-116 colon cancer cells and in a mouse model [77].

Glycolipids have also been shown to be involved in growth arrest and apoptosis of several cancer cells [7,79]. Some glycolipids obtained from marine sponges have been reported, such as α-galacosylceramide (KRN7000) [80,81,82,83], myrmekioside or trikentroside [84]. KRN7000 is a glycolipid containing a sugar moiety connected to a fatty acid chain and a sphingosine base that exhibited anti-cancer activity against liver, lung, EL-4 lymphoma, colon 26 adenocarcinoma, EL-4T cell lymphoma and sarcoma [80,82]. KRN7000 was shown to activate invariant natural killer T cells and subsequent production of interferon (IFN)-g, IL-4 and other cytokines in a dose-dependent manner [81]. Moreover, this glycolipid was found to have an inhibitory effect in advanced cancer patients with hepatitis B or C infection [83]. Myrmekioside is a glycolipid produced by the marine sponge *Myrmekioderma dendyi*. Its derivatives (E-1, E-2 and E-3) exhibited anti-cancer activity against two human non-small-cell lung cancer cells (NSCLC-N6 and A549) [84]. The related glycolipid, trikentroside, isolated from the sponge *Trikentrion,* also inhibited the proliferation of human non-small lung cancer A549 cells [84].

Many biosurfactant chemical structures and anti-cancer activities derived from the marine environment are still to be discovered opening up fascinating opportunities of further developments that certainly will benefit mankind.

## 4. New Perspectives for the Discovery of Novel Therapeutics

Oceans, which cover more than 70% of the Earth’s surface, are one of the richest sources of potentially new bioactive compounds in the world. It is estimated that they contain up to 10^6^ to 10^9^ microbial cells per millilitre of water. As previously mentioned, microbial communities inhabiting marine environments are usually exposed to extreme conditions such as low or high temperatures (0 to 100 °C), high pressures (up to 100 MPa) and low nutrient availability. Their adaption to a broad variety of conditions led to the development of different survival strategies and unique biochemical, metabolic and physiological features, stemming from their wide microbial diversity. Furthermore, as the environmental conditions of marine ecosystems are very different from terrestrial ones, it is expected that marine microorganisms produce compounds with new properties and biological activities comparing to those produced by microbes of terrestrial origin [26,85,86,87].

In the last years, there has been an increasing interest in the study and exploration of marine microorganisms as a source of new compounds for application in different fields. For instance, several biosurfactants with pharmaceutical and medical relevance have been obtained from microorganisms isolated from marine environments, as mentioned in the sections above. However, the recent use of molecular techniques to study the diversity of marine ecosystems revealed that most of the marine microbial world remains unexplored, particularly due to the difficult of growing most of those microorganisms under laboratory conditions [26,85,86]. Culture-independent techniques (metagenomics) are a promising way to study the genetic resources of otherwise inaccessible marine microorganisms, and discover previously unknown natural compounds with important biological activities, without the requirement of culturing them. Metagenomics is a collection of molecular techniques that allow the culture-independent study of microbial communities from any environmental sample through the direct extraction and study of their genetic material, giving access to the total genetic pool and biosynthetic capacity of all the microorganisms present in that community [86,88].

Metagenomic studies usually start with the extraction of the DNA from the environmental sample under study (in the case of marine ecosystems, it can be seawater, marine sediments or marine macro-organisms), although they can be based also on RNA. The DNA sample must represent (both qualitatively and quantitatively) all the microbial species present in the community. The next step is the construction of metagenomic libraries, using suitable cloning vectors. Different vectors can be used, depending on the size of the DNA fragments obtained: plasmids (which can incorporate DNA inserts up to 15 kb); cosmids and fosmids (between 15 and 45 kb); and bacterial artificial chromosomes (up to 100–200 kb). Subsequently, the metagenomic library is transferred to a suitable host strain, usually *E. coli*. Finally, the individual recombinant clones are screened. The screening process can be based on the sequences of the DNA inserts cloned (sequence-based metagenomics), or in the functions that those DNA inserts confer to the host (function-based metagenomics) [85,87,88].

The sequence-based metagenomics can be performed through large-scale random sequencing of metagenomic libraries, which generates a high amount of sequences. Those sequences are subsequently analysed using bioinformatics tools and are compared with sequences deposited in the databases through homology-based searches. Some of those sequences can correspond to novel genes with unknown functions. The development of next-generation sequencing technologies in recent years, as well as the subsequent significant reduction of DNA sequencing costs led to considerable progress in sequence-based metagenomics, allowing its widespread use. On the other hand, sequence-based metagenomics can be performed based on the sequence similarity, using Polymerase Chain Reaction (PCR) or DNA hybridization techniques. In this case, once selected the target genes (or proteins), PCR primers or DNA probes are designed according to consensus sequences specific to the most conserved regions of those genes (or proteins), and these are further used to screen the metagenomic library. The main drawback of this approach is that it requires a previous knowledge of the sequences of the genes, which naturally limits the discovery of new functions or activities [26,85]. For instance, in the specific case of biosurfactants, this approach can be used to search for new genes involved in their biosynthesis based on the sequences of already known genes. As a result, the new biosurfactants found are expected to be similar to those previously reported. However, in some cases, those genes can show slight modifications that can lead to the production of biosurfactants with different properties and activities.

On the contrary, function-based metagenomics consists in screening metagenomic libraries for the presence of activities or phenotypes resulting from the heterologous expression of the genes present in the microbial community. The main advantage of this approach is that it is not dependent on the previous knowledge of the DNA sequences; consequently, it is the best way to identify new genes and gene families encoding novel biomolecules that could not be detected using the sequence-based approaches from comparisons with previously described genes. However, function-based metagenomics entails several difficulties and challenges. Its success is dependent on the expression of the foreign genes in the heterologous host used. The most common host used for the construction of metagenomic libraries is *E. coli*. However, it does not guarantee the expression of all the genes existent in the microbial community under study, due to problems associated with the recognition of promoters from different taxonomic groups by the transcriptional machinery of *E. coli*, or differences in the codon usage preferences. Some examples of functional screening of metagenomic libraries for new antimicrobial compounds using *E. coli* and other alternative hosts simultaneously (*Bacillus subtilis*, *Ralstonia metallidurans* or *Streptomyces lividans*) resulted in the detection of antimicrobial activities in the alternative hosts, but not in *E. coli* [89,90,91]. For that reason, there is a growing tendency to use various hosts simultaneously with the objective of expressing most of the genes present in the microbial community. In this case, the use of broad-host range vectors is necessary to construct the metagenomic libraries. Furthermore, if a function is encoded by several genes grouped in one operon, only if the complete operon is cloned in a single DNA insert could it be detected. For instance, the genes involved in the biosynthesis of lipopeptide biosurfactants (e.g., surfactin, lichenysin, fengycin or iturin), are grouped in operons ranging from 25 to 40 kb in size [92]. This means that the vectors used to construct the metagenomic libraries to identify new biosurfactants must be able to incorporate DNA inserts larger than 20 to 30 kb. Another problem is that, even if the corresponding gene is correctly expressed, if the resulting product is not excreted, it would not be detected during the screening processes. These difficulties are not applicable to the sequence-based metagenomics, where the expression of the DNA inserts is not necessary.

Furthermore, function-based metagenomics requires the development of adequate screening methods that must be sensitive and, at the same time, applicable to thousands of transformants, as metagenomic libraries with sufficient coverage of the whole community require an extremely large number of clones. It can be performed by heterologous complementation of host strains or mutants by target genes to allow their growth under selective conditions, or to allow the use of a specific substrate; or using chromogenic or fluorescent substrates to detect specific enzymatic activities [85,86,87]. For the identification of new antimicrobial compounds, it can be performed by screening clones for antimicrobial activity against clinically relevant microorganisms.

Despite all these issues, there are several examples of new bioactive compounds (mainly enzymes) obtained from marine samples through the application of function-based metagenomics (as reviewed by Felczykowska *et al.* [85] and Kennedy *et al.* [86]). Also, new antimicrobials have been discovered through functional screening of soil metagenomic libraries (as reviewed by Coughlan and co-workers [88]). However, to date there are no reports on the discovery of new biosurfactants using function-based metagenomics, which can be due to the difficulty of establishing an appropriate screening method. Measuring the surface tension or the emulsifying activity is not feasible to screen thousands of transformants. Other techniques commonly used to screen biosurfactant-producing microorganisms, such as oil spreading or the drop collapse assays, can show poor efficiency when applied on a large scale. Furthermore, all these techniques require growing the transformants in liquid medium to evaluate the production of biosurfactants. Several colorimetric techniques can be applied to detect biosurfactants on agar plates, but they are directed to specific biosurfactants, thus they are not useful to identify new compounds. One of these methods uses bromothymol blue (colour indicator) and cetylpyridinium chloride (mediator), which change from yellow-green to dark green or bright blue in the presence of different concentrations of surfactin; it has been successfully used to identify surfactin-hyperproducing *B. subtilis* strains [93]. The cetyltrimethylammonium bromide (CTAB) agar plate method is a semi-quantitative assay for the detection of extracellular anionic biosurfactants. In this case, the potential biosurfactant-producing clones are grown in agar plates containing the cationic surfactant CTAB and the basic dye methylene blue. Biosurfactant-producing colonies are surrounded by dark blue halos due to the formation of an insoluble ion pair between the anionic biosurfactant, CTAB and methylene blue [94]. However, it is not clear if these techniques can be useful to identify new biosurfactants. A more interesting option could be the atomized oil assay described by Burch *et al.* [95], which can be applied directly on agar plates. It consists in applying a fine mist of oil droplets onto the plate (where the transformants have been grown previously) using an airbrush. Biosurfactant production can be detected instantaneously due to the formation of halos around biosurfactant-producing colonies. This technique can detect low concentrations of different chemical surfactants and biosurfactants (including glycolipids and lipopeptides) and is more sensitive than the drop collapse assay [95], thus it could be a good method to screen metagenomic libraries for novel biosurfactants.

## 5. Conclusions

In recent years, there has been an increasing interest in the study and exploration of marine microorganisms as potential producers of new compounds for application in different areas, as the marine environment represents a promising source of novel bioactive compounds. Biosurfactants of marine origin, although less explored than their terrestrial counterparts, exhibit some properties that make them useful and powerful for several therapeutic applications, as alternatives to the existing drugs. However, the difficulty of isolating and growing these marine microorganisms means that most of the marine microbial world remains unexplored. Function-based metagenomics, while exhibiting several limitations, constitutes a promising approach to study the genetic resources of otherwise inaccessible marine microorganisms, giving access to the total genetic pool and biosynthetic capacity of all the microorganisms in a community, as well as allowing the discovery of novel biosurfactants.

## Figures and Tables

**Table 1:** Biosurfactants produced by marine microorganisms with antimicrobial, anti-adhesive or anti-biofilm activities against human pathogens.

Microorganism (Origin)	Biosurfactant Type (Structure)	Activity	Reference
*Brevibacterium casei* MSA19 (marine sponge *Dendrilla nigra*)	Glycolipid (unknown)	Antimicrobial activity against *Escherichia coli*, *Klebsiella pneumoniae*, *Proteus mirabilis*, *Pseudomonas aeruginosa*, haemolytic *Streptococcus*, *Vibrio parahaemolyticus* and *Vibrio vulnificus*	[27]
		Anti-biofilm activity against mixed and individual cultures of *E. coli*, *P. aeruginosa* and *Vibrio* spp.	
*Serratia marcescens* (hard marine coral *Symphyllia* sp.)	Glycolipid (glucose + palmitic acid)	Antimicrobial, anti-adhesive and anti-biofilm activity against *Candida albicans* and *P. aeruginosa*	[28]
*Streptomyces* sp. B3 (marine sediment samples)	Glycolipid (unknown)	Antimicrobial activity against *C. albicans*, *E. coli*, *P. aeruginosa* and *Staphylococcus aureus*	[29]
*Streptomyces* sp. MAB36 (marine sediment samples)	Glycolipid (unknown)	Antimicrobial activity against *Aspergillus niger*, *Bacillus cereus*, *C. albicans*, *Enterococcus faecalis*, *Shigella boydii*, *Shigella dysenteriae* and *S. aureus*	[30]
*Aspergillus ustus* MSF3 (marine sponge *Fasciospongia cavernosa*)	Glycolipoprotein (unknown)	Antimicrobial activity against *C. albicans*, *E. faecalis*, *E. coli*, *K. pneumoniae*, *Micrococcus luteus*, *P. mirabilis*, *P. aeruginosa*, *S. aureus*, *Staphylococcus epidermidis* and haemolytic *Streptococcus*	[31]
*Bacillus circulans* (seawater sample)	Lipopeptide (unknown)	Antimicrobial activity against *Acinetobacter calcoaceticus*, *Citrobacter freundii*, *Enterobacter cloacae*, *E. coli*, *Micrococcus luteus*, *P. mirabilis*, *Proteus vulgaris*, *Serratia marcescens* and multi-drug resistant *E. coli* ^a^, *K. pneumoniae* ^b^ and *S. aureus* ^c^	[32,33]
		Anti-adhesive and anti-biofilm activities against *C. freundii*, *E. coli*, *P. vulgaris*, *Salmonella typhimurium* and *S. marcescens*	
*Bacillus circulans* DMS-2 (marine samples)	Lipopeptide (Mixture of three different Fengycins: β-hydroxy fatty acid of 15, 16 or 17 carbons + cyclic decapeptide)	Antimicrobial activity against *C. freundii*, *E. coli*, *P. vulgaris* and *S. marcescens*	[34]
*Bacillus licheniformis* NIOT-AMKV06 (marine sponge *Acanthella* sp.)	Lipopeptide (unknown)	Antimicrobial activity against *E. faecalis*, *K. pneumoniae*, *M. luteus*, *P. mirabilis*, *Salmonella typhi*, *Shigella flexineri*, *S. aureus* and *Vibrio cholera*	[35]
*Brevibacterium aureum* MSA13 (marine sponge *Dendrilla nigra*)	Lipopeptide (Brevifactin: Octadecanoic acid methyl ester + pro-leu-gly-gly)	Antimicrobial activity against *C. albicans*, *E. coli*, *K. pneumoniae*, *M. luteus*, *P. mirabilis*, *P. aeruginosa*, *S. aureus*, *S. epidermidis* and haemolytic *Streptococcus*	[36]
*Nocardiopsis alba* MSA10 (marine sponge *Fasciospongia cavernosa*)	Lipopeptide (unknown)	Antimicrobial activity against *C. albicans*, *E. faecalis*, *K. pneumoniae*, *M. luteus*, *P. mirabilis*, *S. aureus* and *S. epidermidis*	[37]
*Nocardiopsis dassonvillei* MAD08 (marine sponge *Dendrilla nigra*)	Unknown	Antimicrobial activity against *S. aureus*, *M. luteus* and multi-drug resistant *E. coli* ^d^, *K. pneumoniae* ^d^, *P. mirabilis* ^d^, *P. aeruginosa* ^d^, *S. typhi* ^d^, *S. epidermidis* ^d^, non-haemolytic *Streptococcus* ^d^ and *V. cholera* ^d^	[38]

^a^ resistant to ciprofloxacin, ofloxacin, levofloxacin, streptomycin, penicillin, ceftazidine, norfloxacin and ofloxacin; ^b^ resistant to ceftriaxone, ciprofloxacin, ofloxacin, levofloxacin, norfloxacin, piperacillin, tazobactam, streptomycin and penicillin; ^c^ resistant to methicillin and streptomycin; ^d^ resistant to chloramphenicol, streptomycin, oxytetracycline, ampicillin and erythromycin.
